# Outcome and clinical features in juvenile myasthenia gravis: A systematic review and meta-analysis

**DOI:** 10.3389/fneur.2023.1119294

**Published:** 2023-03-08

**Authors:** Yangtao Lin, Qianjin Kuang, Hongjin Li, Bo Liang, Jiaxin Lu, Qilong Jiang, Xiaojun Yang

**Affiliations:** The First Affiliated Hospital of Chinese Medicine, Guangzhou University of Chinese Medicine, Guangzhou, China

**Keywords:** juvenile myasthenia gravis, clinical features, treatment outcome, autoimmune comorbidity, systematic review, meta-analysis

## Abstract

**Background:**

Juvenile myasthenia gravis (JMG) is a rare autoimmune disease that has so far only been described in small cohort studies. We defined the clinical characteristics, management, and outcomes of JMG patients over the past 22 years.

**Methods:**

A search of PubMed, EMBASE, and web of science (1/2000–2/2022) identified all English language and human studies of JMG. The population was patients diagnosed with JMG. Outcomes included the history of myasthenic crisis, autoimmune comorbidity, mortality, and treatment outcome. Data extraction was performed by independent reviewers. And we performed a pooled reanalysis of all published data in the included studies and compared with other studies of adult cohorts.

**Results:**

We identified 11 articles describing 1,109 patients diagnosed between 2006 and 2021. JMG occurred in 60.4% of female patients. The mean age at presentation was 7.38 years old, and 60.6% of the patients had ocular symptoms as the first clinical manifestation. The most common initial presentation was ptosis, which occurred in 77.7% patients. AchR-Ab positive accounted for 78.7%. 641 patients received thymus examination, found to have thymic hyperplasia in 64.9% and thymoma in 2.2%. Autoimmune comorbidity was found in 13.6% and the most common one is thyroid disease (61.5%). First-line therapy, including pyridostigmine and steroids, was initiated in 97.8 and 68.6%, respectively. Six patients resolved spontaneously without treatment. Thymectomy was performed in 45.6%. 10.6% of patients had a history of myasthenic crisis. Completely stable remission was achieved in 23.7% and mortality was reported in 2 studies, which reported 8 deaths.

**Conclusion:**

JMG is a rare disease with a relatively benign course, and differs from adult MG in some clinical features. The treatment regimen guideline for children is still not well-established. There is a need for prospective studies to properly evaluate treatment regimes.

## Background

Juvenile myasthenia gravis (JMG) is an autoimmune disorder that leads to dysfunction of acetylcholine receptors (AchR), defined as myasthenia gravis in children younger than 18 years of age (1). It is unclear whether the pathogenesis of JMG is the same as that of adults. While clinical phenotypes are similar to adults (2), JMG and adult MG still have many different characteristics, such as symptoms, clinical severity, antibody titer, and thymus histology (3). Current practice is taken from treatment guidelines for adult MG or individual experience, and it still has no standardized treatment guidelines (4, 5).

We performed a systematic review and meta-analysis to determine the clinical features, treatment, and outcomes of patients with JMG over the last 22 years.

## Methods

### Search strategy

PubMed, EMBASE, and Web of science were searched using the search topic “juvenile myasthenia gravis”. Given that the earlier articles did not collect enough clinical data, articles published between January 2000 and February 2022 were included. Limiters of human studies, English language, non-case reports and non-case series studies were applied. This search yielded 181 studies. The titles and abstracts for the identified manuscripts were evaluated per the inclusion/exclusion criteria described below. Reference lists for relevant review articles were searched manually for additional studies. In addition, topic experts were contacted to determine if any additional studies or unpublished data could be identified. The titles and abstracts for all included publications were assessed by two independent reviewers, resulting in 11 full-text articles (6–16), which were subsequently evaluated in further details.

Inclusion/exclusion criteria

Studies were evaluated using inclusion criteria, as follows:

1. Patients diagnosed with JMG (clinical presentation consistent with JMG as well as elevated serum antibodies, positive stimulatory test, or response to a trial of therapy);
2. Onset < 18 years of age;
3. Intervention of standard treatment;
4. Collected enough clinical data including the age of onset, sex, symptom, serology, and treatment outcome.
5. Patients >10.

The decision for the inclusion of each study was made independently by 2 independent reviewers. Disagreements between reviewers were resolved by consensus. Data extraction focused on study methodology, population, intervention, results. Extracted data included: age of onset (pre-pubertal ≤ 12 years, post-pubertal>12 years), sex, symptom of onset, seropositivity, results for repetitive nerve stimulation (RNS) and single fiber electromyography (SFEMG), thymic pathology, myasthenic crisis, autoimmune comorbidity, generalization, treatment and treatment outcome. Data extraction was performed using standardized tables, which were subsequently corroborated and synthesized.

Risk of bias was assessed using the criteria described in the Newcastle-Ottawa Scale by two authors independently. For all cohort studies, the assessments included methods of selecting exposure and non-exposure cases, comparability between groups, and evaluation of outcome incidents. This process was performed independently by two investigators. Any disagreement between investigators was resolved by reconciliation and/or discussion with a third investigator (Results shown in Supplementary file 1).

We performed a pooled reanalysis of all published data in the included studies and compared with other studies of adult cohorts. Because of heterogeneity between studies, all data are presented as the proportion (n/N [%]) of the total number of patients with a certain characteristics and analyzed with 95% confidence intervals (95% CI). Heterogeneity between studies was calculated for all reported variables using the I^2^ statistical tests by Stata 16.0 (Supplementary file 1).

## Results

**Figure 1:**
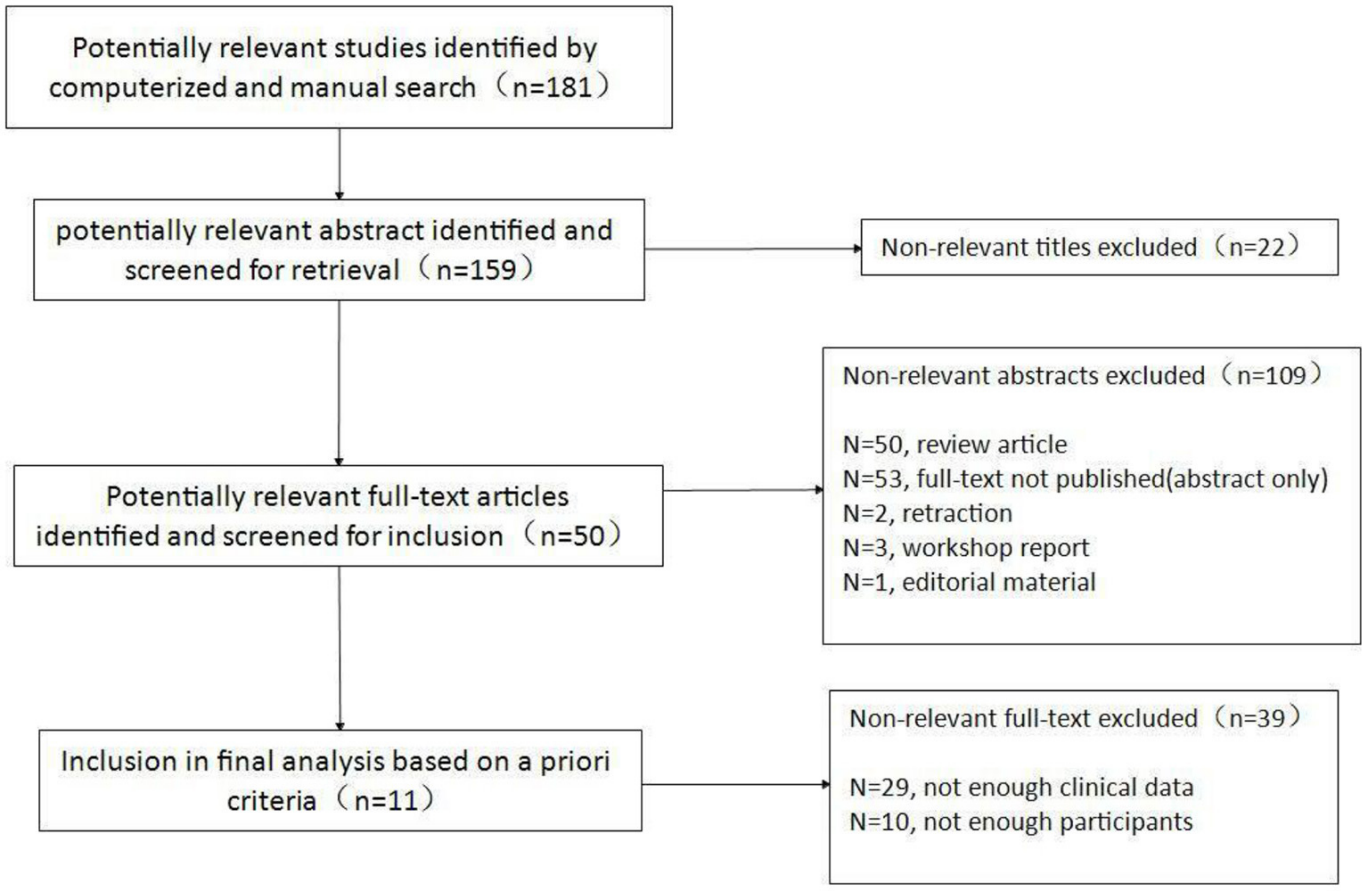
Flow chart of inclusion of studies.

Our search identified 181 articles, of which 11 studies remained after screening (Figure 1), covering 1,109 patients diagnosed between 2006 and 2021. All studies were retrospective studies (Table 1). The number of patients in each study varied between 40 and 327. Participants were followed for a period ranging from 0.2 to 67 years. Heterogeneity calculations for the data reported in pooled study results revealed significant heterogeneity (I^2^>50%) for 24 of the 40 study parameters (Supplementary file 1).

**Table 1:** Characteristics of included studies.

**Author**	**Year**	**Region**	**Study design**	**Number of patients**	**Mean follow-up time**
Popperud et al. (6)	2021	Europe	Cohort	47	12 y
Asenova and Bojinova (7)	2020	Europe	Cohort	43	NA
Jastrzebska et al. (8)	2019	Europe	Cohort	101	12.8 y
Vanikieti et al. (9)	2018	Asia	Cohort	62	7.9 y
Huang et al. (10)	2018	Asia	Cohort	327	≥1 y
Barraud et al. (11)	2018	Europe	Cohort	40	4.7 y
Wejwittayaklung et al. (12)	2017	Asia	Cohort	71	3 y
Popperud et al. (13)	2017	Europe	Cohort	63	14.8 y
Lee et al. (14)	2016	Asia	Cohort	88	NA
Heckmann et al. (15)	2012	Africa	Cohort	190	4 y
Ashraf et al. (16)	2006	Asia	Cohort	77	6.2 y

### Patient characteristics

These patients were from three main regions, including Asia (56.4%), Europe (26.5%) and Africa (17.1%). The majority of patients were female (*n* = 670, 60.4%; 95% CI 55–72%). The mean age at presentation was 7.38 years (ranging from 1 to 216 months). It is reported that 664 (64.3; 95% CI 44–77%) patients had pre-pubertal onset, and 638 patients (35.7%) had post-pubertal onset (Table 2).

**Table 2:** Patient characteristics.

**Characteristic**	**n/N (%)**	**Characteristic**	**n/N (%)**
Region		Thymic hyperplasia	351/541 (64.9)
Europe	294/1,109 (26.5)	Thymoma	11/509 (2.2)
Asia	625/1,109 (56.4)	Thymic atrophy	4/509 (0.8)
Africa	190/1,109 (17.1)	Normal	183/509 (36.0)
Age of onset, yr., mean	7.38	Myasthenic crisis	108/1,019 (10.6)
Pre	664/1,032 (64.3)	Autoimmune comorbidity	113/830 (13.6)
Post	368/1,032 (35.7)	Thyroid disease	59/96 (61.5)
Sex, female	670/1,109 (60.4)	Hypothyroidism	12/48 (25.0)
Symptom of onset		Hyperthyroidism	34/48 (70.8)
Ocular	672/1,109 (60.6)	Hashimoto thyroiditis	2/48 (4.2)
Generalized	437/1,109 (39.4)	Nephrosis	1/85 (1.2)
Seropositivity		SLE	4/85 (4.7)
AchR Ab	571/726 (78.7)	ITP	2/85 (2.4)
MUSK Ab	5/146 (3.4)	Mb Crohns/UC	3/85 (3.5)
RNS		Juvenile rheumatoid arthritis	3/85 (3.5)
Positive	189/241 (78.4)	Vitiligo	4/85 (4.7)
SFEMG		Psoriasis	5/85 (5.9)
Abnormal	56/78 (71.8)	Alopecia areata	1/85 (1.2)
Thymic pathology		Type 1 diabetes mellitus	3/85 (3.5)

AchR, acetylcholine receptor; MUSK, muscle-specific tyrosine kinase; RNS, Repetitive nerve stimulation; SFEMG, single fiber electromyography; SLE, systemic lupus erythematosus; ITP, idiopathic thrombocytopenic purpura; UC, Ulcerative colitis.

### Clinical characteristics

Ocular symptoms were the first clinical manifestation of JMG in 672 of 1,109 patients (60.6%; 95% CI 31–82%), including ptosis, diplopia, and strabismus. And 437 of 1,109 patients (39.4%) presented with generalized symptoms at first. The most common initial presentation was ptosis, which occurred in 422 of 543 patients (77.7%; 95%CI 65–85%). At presentation, 64 of 769 patients (8.3%) had limb weakness and 55 of 732 patients (7.5%) had bulbar weakness. Seven of 492 patients (0.1%) developed respiratory muscle weakness.

In these researches, 571 of 726 (78.7%; 95% CI 65–85%) patients with antibody status were AchR-Ab positive. Among 146 cases of muscle-specific kinase (MUSK) antibody test results, 5 cases (3.4%) were positive. RNS test showed a positive decrement in 189 of 241 (78.4%; 95% CI 56–88%) patients. And SFEMG was abnormal in 56 of 78 (71.8%) patients.

**Figure 2:**
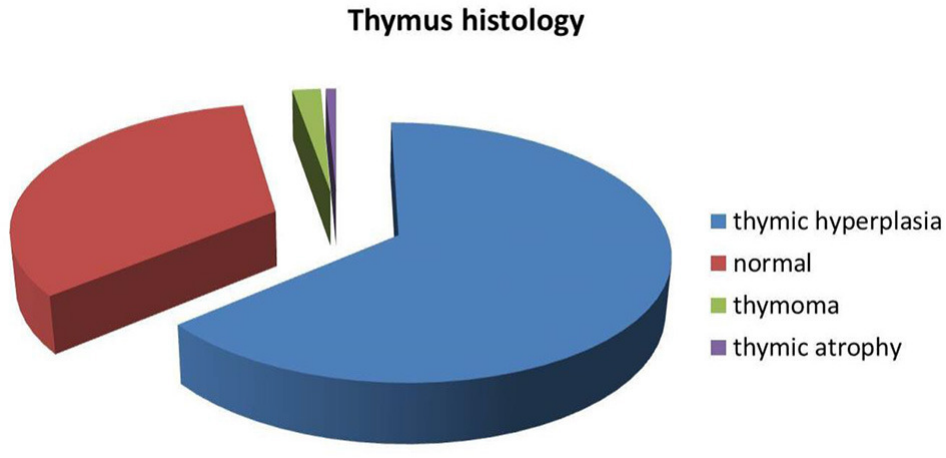
Proportion of thymic pathology.

In these researches, 541 patients underwent computed tomography (CT)/magnetic resonance imaging (MRI)/ thymus biopsy to investigate thymic pathology. The most common histopathology was thymic hyperplasia which was detected in 351 of 541 (64.9%; 95% CI 41–76%) patients. Thymic atrophy was the rarest histopathology, found in 4 of 509 (0.8%) patients; 11 of 509 (2.2%; 95% CI 0–3%) patients had thymoma and 183 of 509 (36.0%; 95% CI 19–65%) patients had a normal thymus (Figure 2).

**Figure 3:**
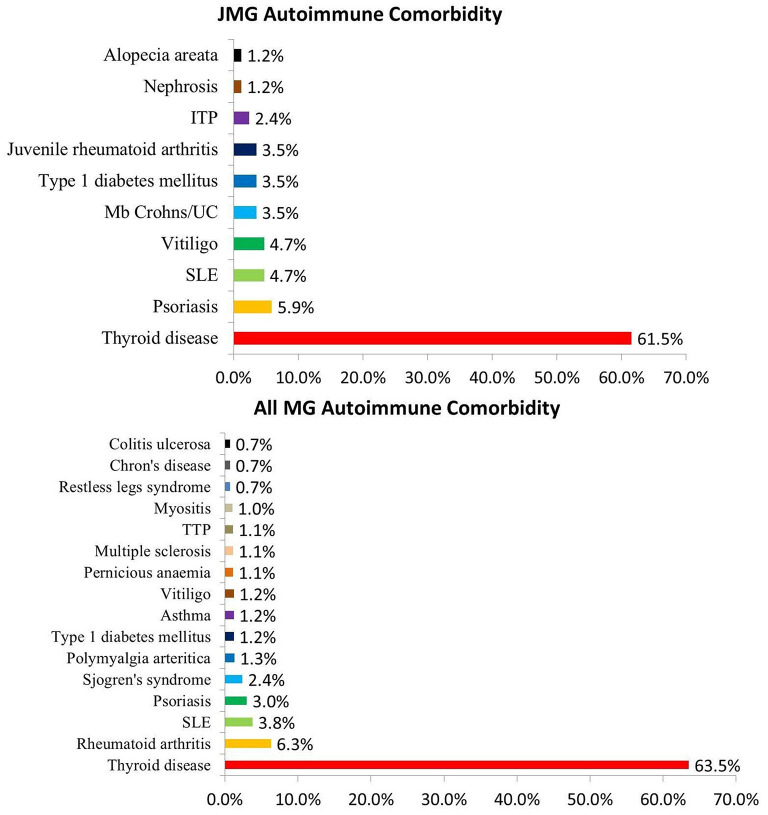
Proportion of autoimmune comorbidity in JMG and MG. Abbreviations: SLE, systemic lupus erythematosus; ITP, idiopathic thrombocytopenic purpura; TTP, Thrombotic thrombocytopenic purpura; UC, Ulcerative colitis.

In these researches, 113 of 830 patients (13.6%; 95% CI 8–24%) were found to have other autoimmune diseases; thyroid disease was the most common (*n* = 59, 61.5%; 95% CI 22–90%). Hyperthyroidism was found in 34 of 48 (70.8%) patients with thyroid disease, while hypothyroidism was found in 12 of 48 (25.0%) patients. And 2 patients had hashimoto thyroiditis. Other autoimmune diseases included nephrosis (1/85), systemic lupus erythematosus (SLE) (4/85), idiopathic thrombocytopenic purpura (ITP) (2/85), Mb Crohns/ Ulcerative colitis (UC) (3/85), juvenile rheumatoid arthritis (3/85), vitiligo (4/85) psoriasis (5/85), alopecia areata (1/85), type 1 diabetes mellitus (3/85) (Figure 3).

### Treatment and outcome

**Figure 4:**
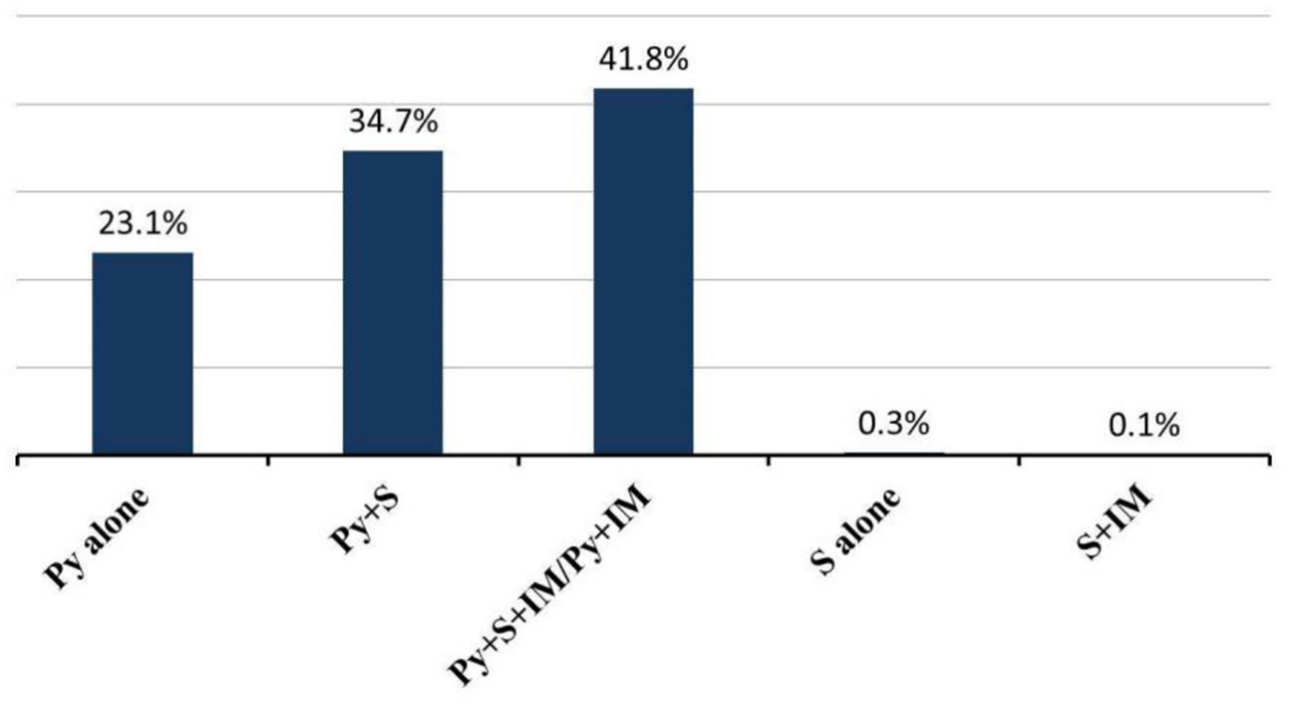
Combination treatment in JMG patients. Abbreviations: Py, pyridostigmine; S, steroids; IM, Immunosuppressants.

Almost all patients were treated with pyridostigmine (97.8%; 95% CI 97–100%), except for patients in spontaneous remission (*n* = 6, 0.6%) without any treatment and 17 patients who received other medications first. First-line therapy, consisting of steroids, was initiated in 729 of 1,062 (68.6%; 95% CI 41–74%) patients; and 356 of 1,019 (34.9%; 95% CI 15–38%) patients were treated with other immunosuppressants, including azathioprine, cyclophosphamide, cyclosporine, methotrexate, mycophenolate mofetil, and rituximab. There were 741 patients reported combination treatment (Figure 4). Intravenous immunoglobulin (IVIg) was given to 71 of 829 (8.6%; 95% CI 1–23%) patients during acute exacerbation or crisis, while plasmapheresis (PE) was given to 40 of 829 (4.8%; 95% CI 1–13%) patients. Thymectomy was performed in 502 of 1,101 (45.6%; 95% CI 19–56%) patients. Among these patients, 167 of 457 (36.5%; 95% CI 0–39%) patients were pure ocular MG and 290 of 457 (63.5%; 95% CI 61–100%) patients were generalized MG (Table 3).

**Table 3:** Treatment and outcome.

**Characteristic**	**n/N (%)**	**Characteristic**	**n/N (%)**
Treatment		Thymectomy	502/1,101 (45.6)
Pyridostigmine	1,039/1,062 (97.8)	OMG	167/457 (36.5)
Steroids	729/1,062 (68.6)	GMG	290/457 (63.5)
Immunosuppressants	356/1,019 (34.9)	Treatment outcome	
IVIg	71/829 (8.6)	CSR	203/856 (23.7)
PE	40/829 (4.8)	PR	54/461 (11.7)
No treatment	6/1,019 (0.6)	MM	216/651 (33.2)
Generalization	126/576 (21.9)	Death	8/1,109 (0.7)

IVIg, Intravenous immunoglobulin; PE, plasma exchange; CSR, completely stable remission; PR, pharmacologic remission; MM, minimal manifestation.

Treatment response is shown in Table 3. Mortality was reported in 2 studies, which reported 8 deaths (0.7%). Six of them died from myasthenic crisis, and the other two died from unrelated reasons-−1of HIV and 1 of ischaemic heart disease. Completely stable remission was achieved in 203 of 856 (23.7%; 95% CI 19–33%) patients, pharmacologic remission in 54 of 461 (11.7%) patients, and minimal manifestation in 216 of 651 (33.2%) patients.

In these researches, 108 of 1,019 (10.6%; 95% CI 4–16%) patients had a history of myasthenic crisis, and most of them were treated successfully with PE or IVIg. And there were 6 deaths in the course of the crisis.

126 of 576 (21.9%; 95% CI 14–44%) patients evolved into generalized MG during the follow-up time. Five studies (9–14) reported time to generalization (ranging from 1 month to 14 years), and there were 55 of 65 (84.6%) patients evolved into generalized MG within 2 years.

## Discussion

Our data show that juvenile myasthenia gravis is a rare autoimmune disease with more common ocular symptoms, and a relatively benign course, and a better prognosis than the adult disease (17). Although the data were not comprehensive enough, it can still summarize some clinical features of JMG.

In our review, a greater proportion of JMG patients had pre-pubertal onset. And pre-pubertal patients tended to have more ocular presentations (14, 15). Seronegative cases were also seen more frequently among them (8, 11). Generally, pre-pubertal JMG patients responded better to treatment than post-pubertal patients (8).

To better show the differences in clinical features between JMG and adult MG, we made a comparison with a large cohort of adult MG (17) in Table 4.

**Table 4:** Comparison of clinical characteristics with JMG and adult MG.

**Patient Characteristics, n/N (%)**	**Juvenile MG**	**Adult MG (17)**
	***N*** = **1,109**	***N*** = **939**
**Gender**		
Male (%)	39.6	52.8
Female (%)	60.4	47.2
**Antibodies**		
AchR	571/726 (78.7)	799/939 (85.1)
MUSK	5/146 (3.4)	25/926 (2.7)
Ocular onset	672/1,109 (60.6)	374/916 (40.8)
Bulbar onset	55/732 (7.5)	360/916 (39.3)
Myasthenic crisis	108/1,019 (10.6)	66/884 (7.5)
Generalization	126/576 (21.9)	114/349 (32.7)
Thymoma	11/509 (2.2)	123/918 (13.4)
**Treatment outcome**		
CSR	203/856 (23.7)	83/939 (8.8)
PR	54/461 (11.7)	300/939 (31.9)
MM	216/651 (33.2)	264/939 (28.1)
Death	6/1,223 (0.7)	114/939 (12.1)

AchR, acetylcholine receptor; MUSK, muscle-specific tyrosine kinase; CSR, completely stable remission; PR, pharmacologic remission; MM, minimal manifestation.

This review shows a female preponderance in the juvenile MG group, but not in the adult group. The fact is that estrogen reduces the expression of the autoimmune regulator gene in the thymus of young women, resulting in increased release of autoreactive T cells (18, 19). Seropositivity was almost similar to that observed in adults, both AchR and MUSK, similar to other studies (8, 16, 20). MUSK antibody positive is rare in both JMG and adult MG. Ocular symptoms were more common in the JMG group (9), but bulbar symptoms were less. The incidence of myasthenic crisis was similar in the two groups. However, the mortality rate of adult MG was significantly higher than that of JMG. It suggests that the cause of death in myasthenia gravis is not solely related to crisis. Thymoma can also lead to serious complications leading to death (19, 21). And thymoma is less common in JMG than in adults. This is one of the reasons for the low mortality in JMG. Consequently, the number of children achieving completely stable remission was also significantly higher than that of adults (9).

In our review, generalization of symptoms occurred in 21.9% of JMG patients. And the rate is lower than in the adult group, even much lower than in other study, where it reaches 50–80% (22, 23). This may explain the higher prevalence of isolated ocular MG among the juvenile population.

**Figure 5:**
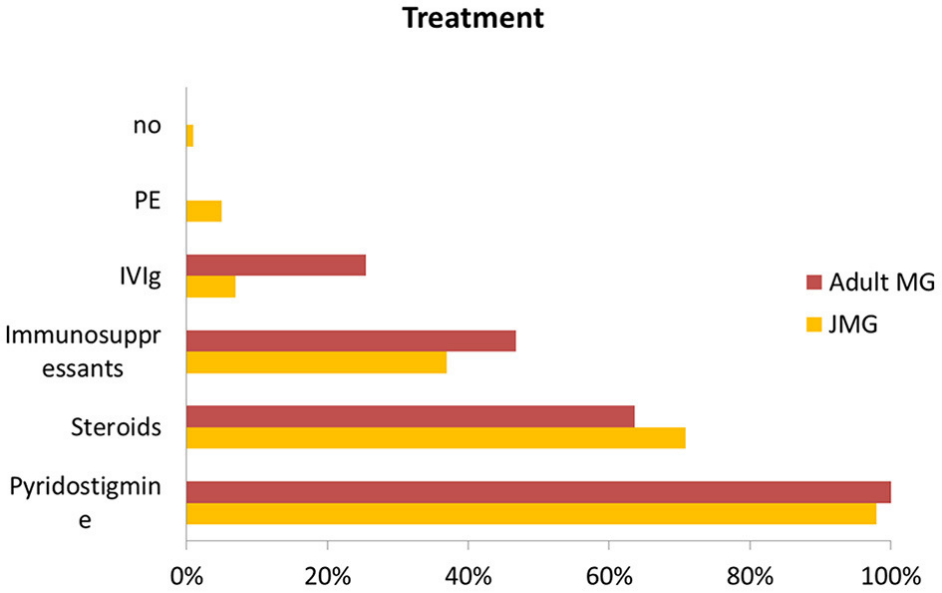
Comparison of treatment with JMG and adult MG. Abbreviations: IVIg, Intravenous immunoglobulin; PE, plasma exchange.

Juvenile MG has a relatively benign course. However, the treatment regimen guideline for children is not well-established. And current practice has been taken from adult guidelines and expert opinion based on individual experience (5, 24, 25). Typically, cholinesterase inhibitors and steroids are used first-line as symptomatic treatment in JMG (4, 26–28). Other immunosuppressants are the second-line therapy for both generalized MG and uncontrolled ocular MG when they failed to respond to anticholinesterase inhibitor therapy (12). Intravenous immunoglobulin (IVIG) and plasma exchange (PE) have also been used as maintenance therapy (29). Compared with the JMG group, adult patients more often received combinations of steroids and other immunosuppressive drugs, also IVIg, (Figure 5) but even so a lower proportion achieved completely stable remission.

In the JMG group, ocular MG and generalized MG have different prognosis. OMG typically has a higher remission rate than GMG (10, 15), and a lower incidence of myasthenic crisis (12). Most OMG patients could achieve remission with pyridostigmine alone, while GMG patients need combination treatment to achieve remission (12).

Autoimmune comorbidity is a known feature in patients with myasthenia gravis, and also in those with juvenile-onset (13, 30, 31). We also collected some data from other studies of MG patients in combination with other autoimmune diseases, and made statistics in Figure 3 (18, 32–35). 1,863 of 18,337 MG patients (10.2%) were found to have other autoimmune diseases, the incidence similar to the JMG group (13.6%). Thyroid disease was the most common autoimmune comorbidity in MG patients (6), also in the JMG group. There was no clear trend toward clustering in other autoimmune diseases (Figure 3).

Thymectomy is an important treatment option for adults with myasthenia gravis when medical therapy is refractory, but remains controversial in the juvenile myasthenia gravis population. Although several recent studies have shown the benefits of thymectomy in JMG patients (6, 36, 37), they are all retrospective and have limitations. And it was reported that some patients presented a crisis after thymectomy, requiring hospitalization and ventilatory support (38).

In our review, 41.8% of JMG patients performed thymectomy, and 36.5% of them were pure ocular MG. Despite the lack of prospective studies evaluating thymectomy in JMG, it is generally accepted that thymectomy is considered as part of the initial management of JMG patients with abnormal thymus pathology, such as thymoma and thymic hyperplasia. Meanwhile, thymectomy can also perform in AchR-Ab positive generalized JMG patients and JMG patients who do not respond well to medication (3). There is still a lack of large prospective cohort studies of JMG patients to standardize the use of thymectomy for JMG.

## Conclusion

Our systematic review and meta-analysis found that JMG has some distinctive clinical features, such as better treatment outcome and lower generalization compared with adult. Nevertheless, it also has limitations. All studies were retrospective, introducing selection bias. JMG is a rare disease, and evidence-based guidelines are lacking. To standardize treatment guidelines, future prospective multicenter studies are needed for the best treatment outcome.

## Data availability statement

The original contributions presented in the study are included in the article/Supplementary material, further inquiries can be directed to the corresponding authors.

## Author contributions

QJ and YL designed the study. YL and QK conducted the literature search, collected, analyzed, interpreted the data, and drafted the first manuscript. HL, BL, and JL interpretation of data and revision of manuscript for intellectual content. QJ analysis and interpretation of data and revision of the manuscript for intellectual content. All authors have read and approved the final version of the manuscript.
