# Supplementary material for: Outcome and clinical features in juvenile myasthenia gravis: A systematic review and meta-analysis
Source: Front Neurol. 2023 Mar 8;14:1119294. doi: 10.3389/fneur.2023.1119294 (PMC10032342; doi:10.3389/fneur.2023.1119294)
Supplement: Supplementary file 1 [file Data_Sheet_1.docx]

**Outcome and clinical features in juvenile myasthenia gravis: a systematic review and meta-analysis**

**Supplementary Table 1** Results of quality assessment using the Newcastle-Ottawa for cohort studies

| **Study** | **Selection** | | | | **Compa-rability** | **Outcome** | | | **Scores** |
| --- | --- | --- | --- | --- | --- | --- | --- | --- | --- |
|  | Representat-iveness of the exposed cohort | Selection of the non exposed cohort | Ascertain-ment of exposure to implants | Demonstr-ation that outcome of interest was not present at start of study |  | Assessm-ent of outcome | Was follow up long enough for outcomes to occur | Adequacy of follow up of cohorts |  |
| Popperud 2021 | ☆ | - | ☆ | - | ☆ | ☆ | ☆ | - | 5 |
| Asenova 2020 | ☆ | - | ☆ | - | ☆ | ☆ | ☆ | - | 5 |
| Jastrzębska 2019 | ☆ | - | ☆ | - | ☆ | ☆ | ☆ | - | 5 |
| Vanikieti 2018 | ☆ | - | ☆ | - | ☆☆ | ☆ | ☆ | ☆ | 7 |
| Huang 2018 | ☆ | - | ☆ | - | ☆ | ☆ | ☆ | ☆ | 6 |
| Barraud 2018 | ☆ | - | ☆ | - | ☆ | ☆ | ☆ | ☆ | 6 |
| Wejwittayaklung 2017 | ☆ | - | ☆ | - | ☆ | ☆ | ☆ | ☆ | 6 |
| Popperud 2017 | ☆ | - | ☆ | - | ☆ | ☆ | ☆ | ☆ | 6 |
| Lee 2016 | ☆ | - | ☆ | - | ☆ | ☆ | ☆ | ☆ | 6 |
| Heckmann 2012 | ☆ | - | ☆ | - | ☆ | ☆ | ☆ | ☆ | 6 |
| Ashraf 2006 | ☆ | - | ☆ | - | ☆ | ☆ | - | ☆ | 5 |

**Supplementary Table 2** Heterogeneity of variables between studies

| **Characteristic** | **I^2^** | **Characteristic** | **I^2^** |
| --- | --- | --- | --- |
| Age of onset |  | Mb Crohns/UC | 0 |
| Pre | 96 | Juvenile rheumatoid arthritis | 0 |
| Post | 96 | Vitiligo | 0 |
| Sex, female | 86 | Psoriasis | 0 |
| Symptom of onset |  | Alopecia areata | 0 |
| Ocular | 99 | Type 1 diabetes mellitus | 0 |
| Generalized | 99 | Treatment |  |
| Seropositivity |  | Pyridostigmine | 77 |
| AchR Ab | 89 | Steroids | 96 |
| MUSK Ab | 9 | Immunosuppressants | 94 |
| RNS |  | IVIg | 96 |
| positive | 85 | PE | 92 |
| SFEMG |  | No treatment | 0 |
| abnormal | 0 | Thymectomy | 98 |
| Thymic pathology |  | OMG | 98 |
| thymic hyperplasia | 94 | GMG | 98 |
| thymoma | 12 | Treatment outcome |  |
| thymic atrophy | 0 | CSR | 79 |
| normal | 96 | PR | 98 |
| Myasthenic crisis | 90 | MM | 64 |
| Autoimmune comorbidity | 89 | Death | 0 |
| Thyroid disease | 89 | Generalization | 92 |
| Nephrosis | 0 |  |  |
| SLE | 0 |  |  |
| ITP | 0 |  |  |

**Supplementary Figures**


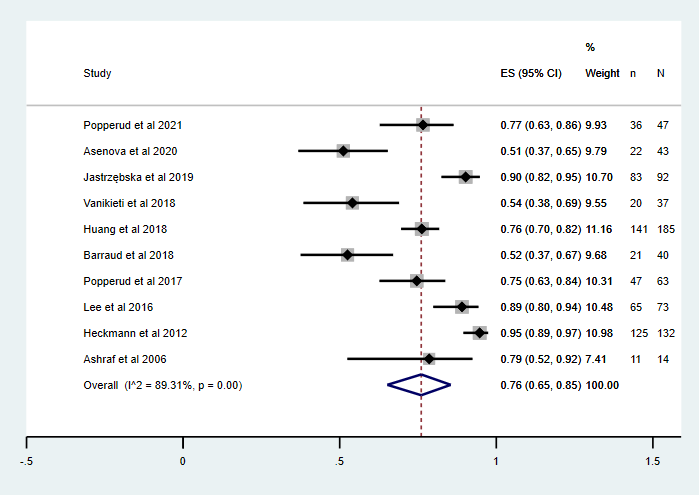


Supplementary fig. 1 JMG with AChR-Ab(+) forest plot


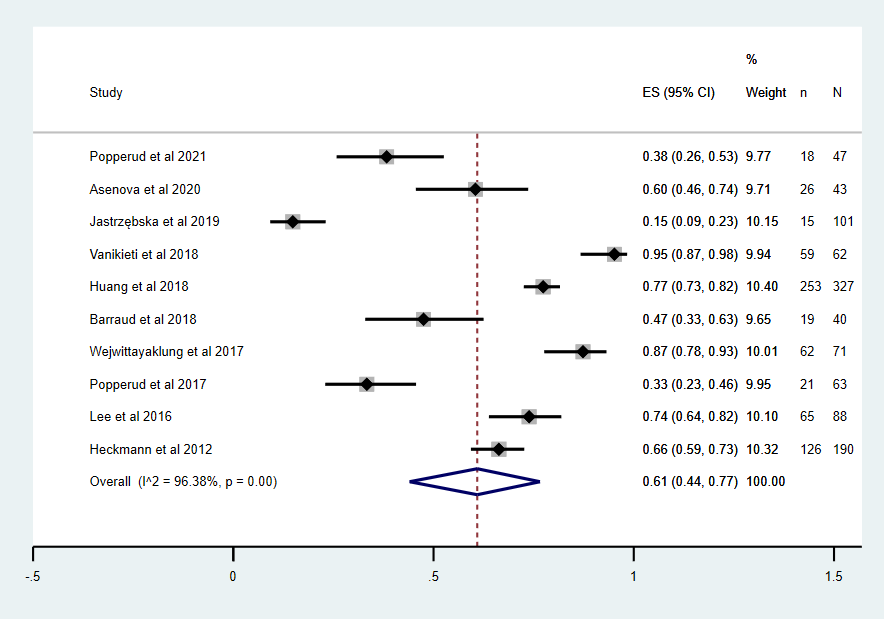


Supplementary fig. 2 JMG pre-pubertal onset forest plot

Supplementary fig. 3 JMG with autoimmune comorbidity forest plot


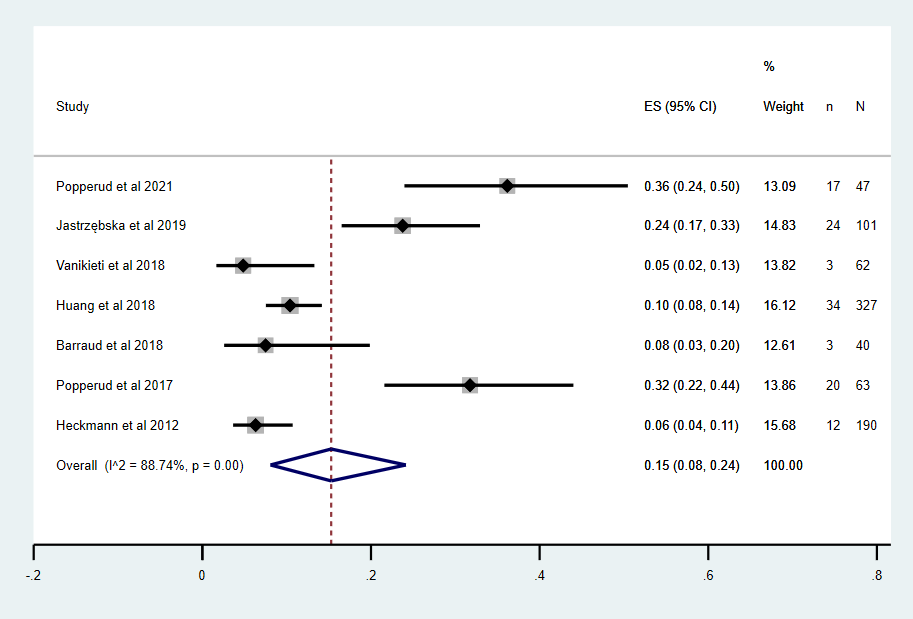


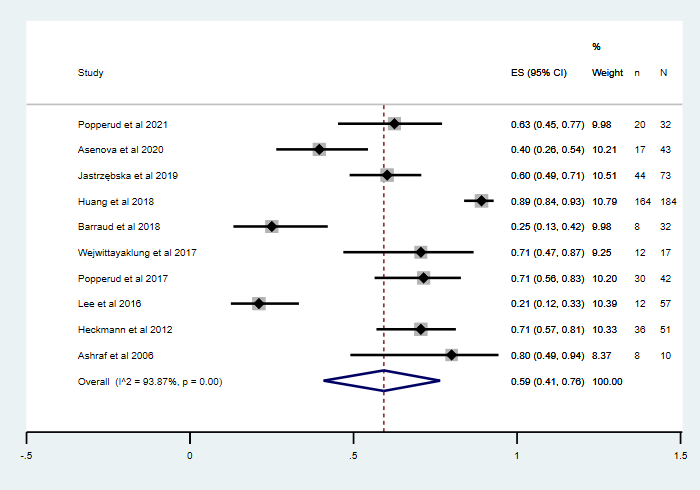


Supplementary fig. 4 JMG with thymic hyperplasia forest plot


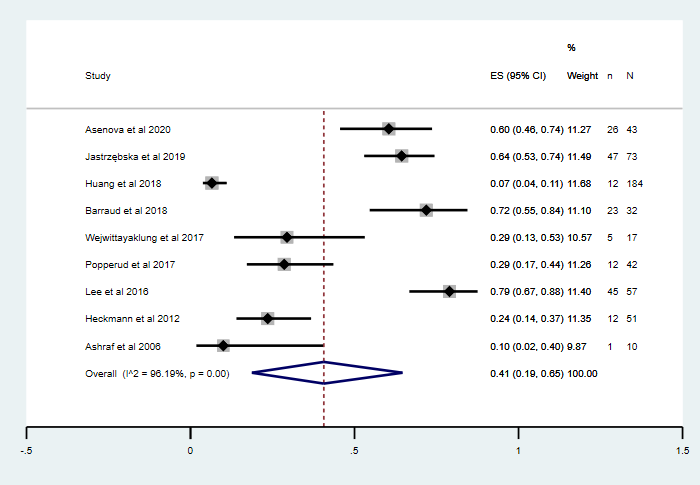


Supplementary fig. 5 JMG with normal thymus forest plot


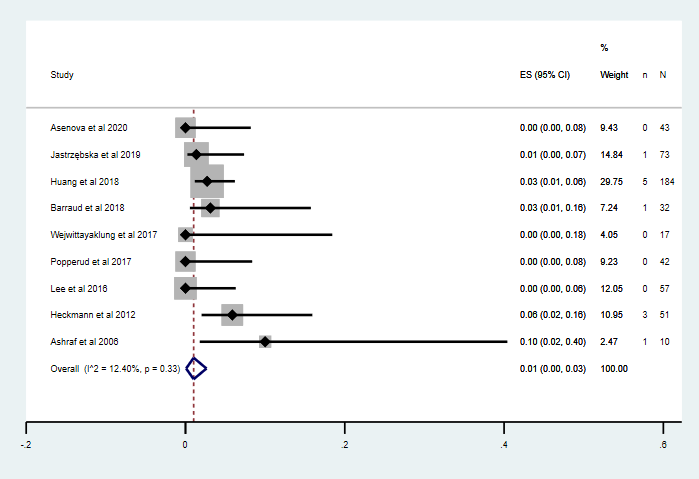


Supplementary fig. 6 JMG with normal thymoma forest plot


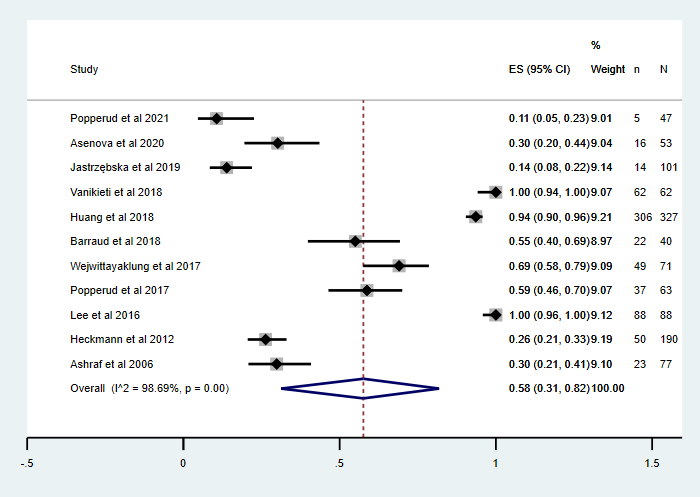


Supplementary fig. 7 JMG ocular symptoms forest plot

Supplementary fig. 8 JMG with ptosis forest plot


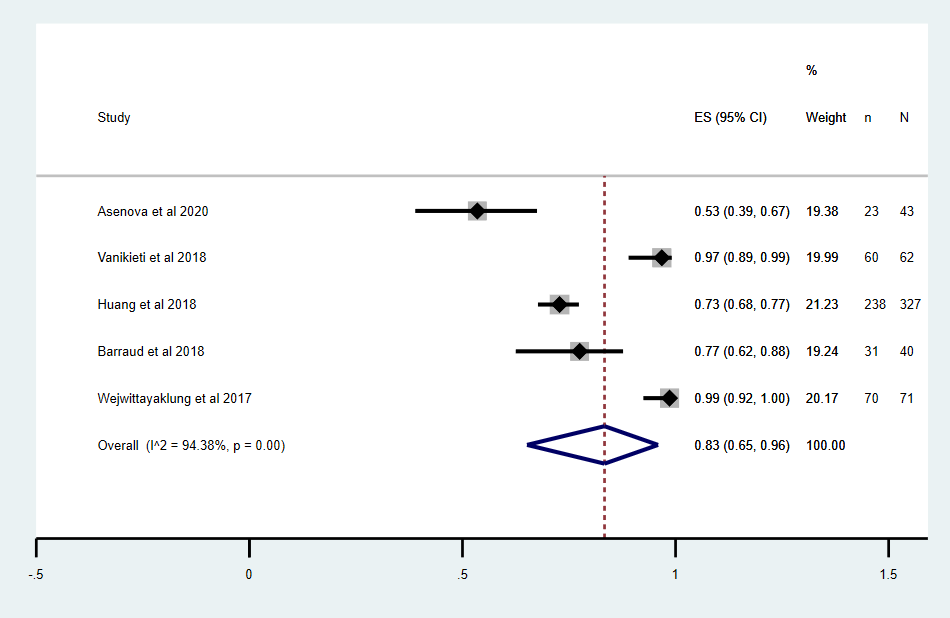


Supplementary fig. 9 JMG with RNS positive forest plot


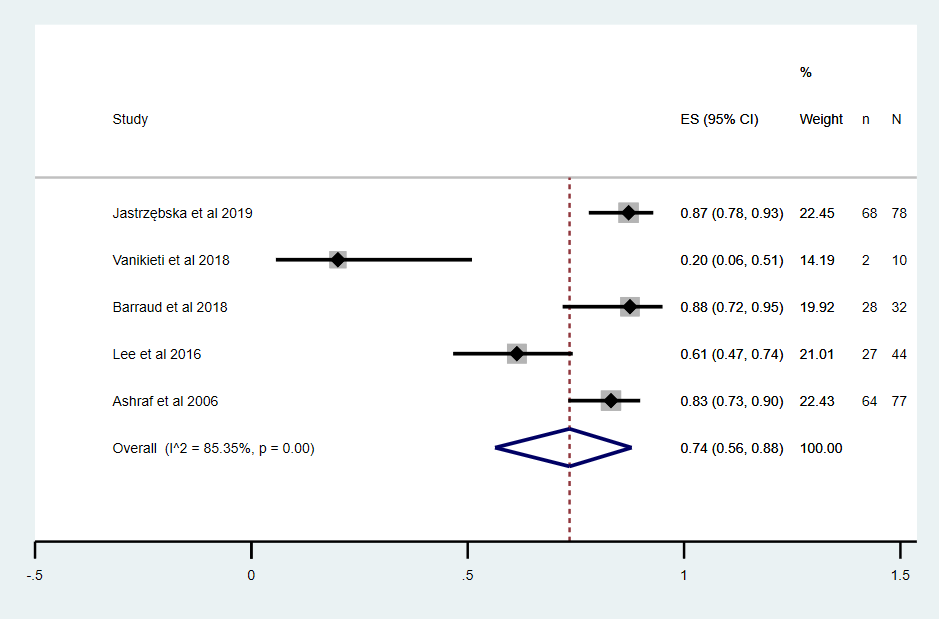


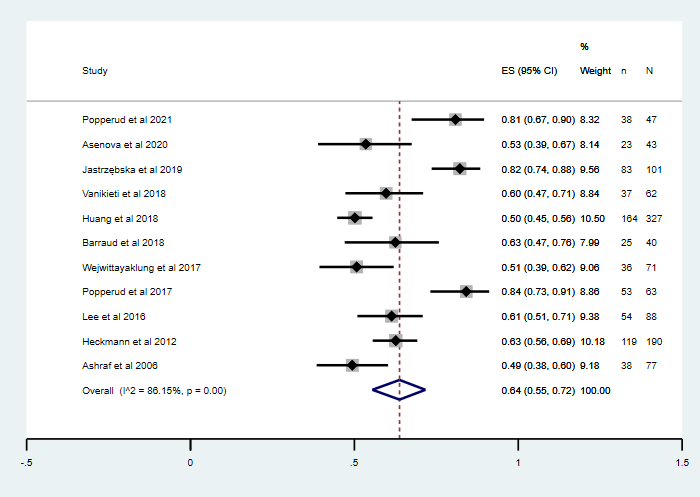


Supplementary fig. 10 JMG female ratio forest plot


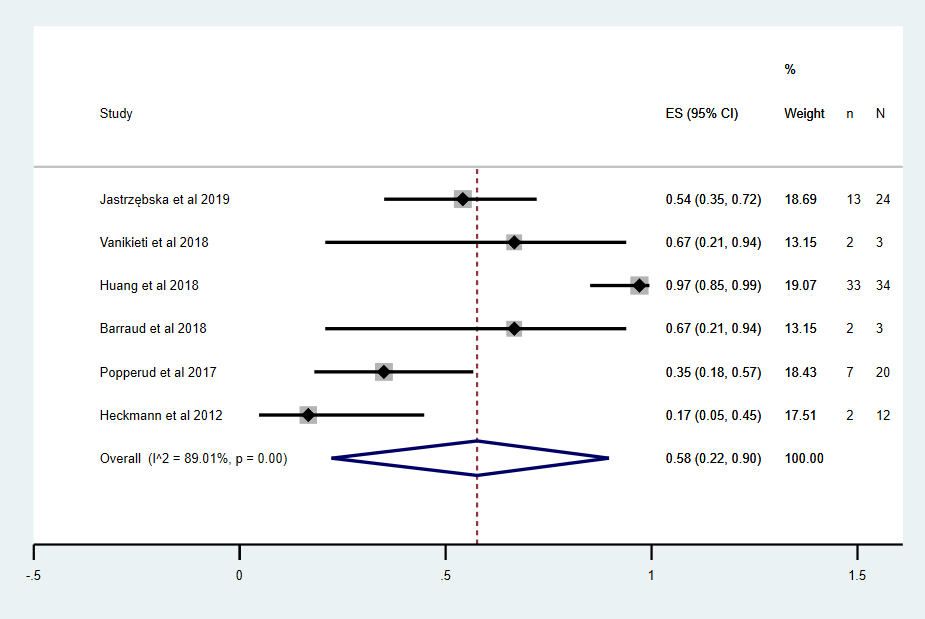


Supplementary fig. 11 JMG with thyroid disease forest plot


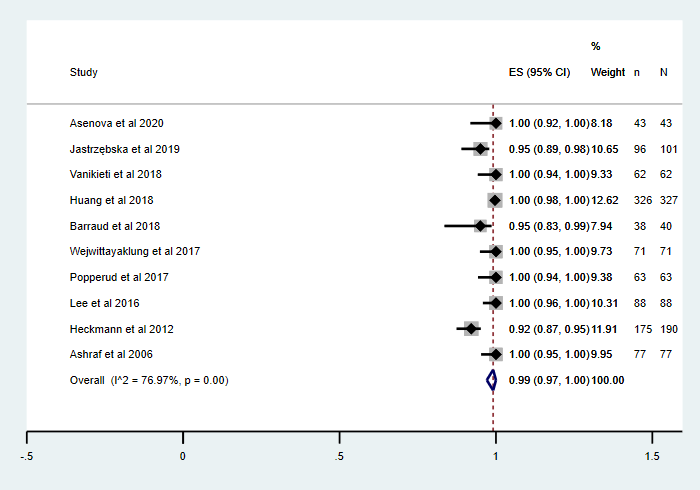


Supplementary fig. 12 JMG treated with pyridostigmine forest plot


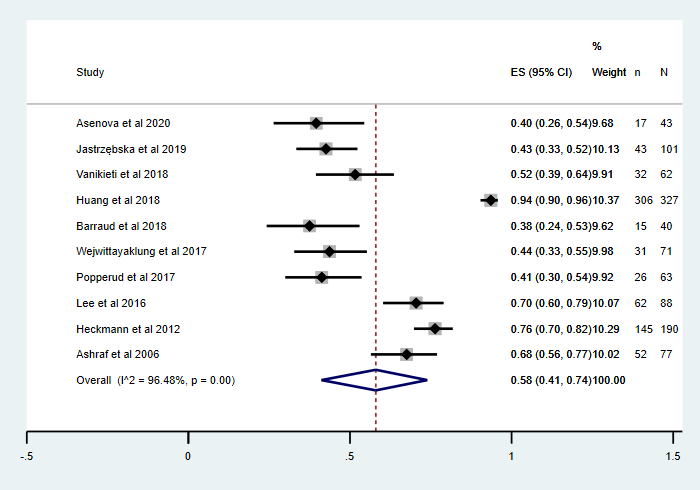


Supplementary fig. 13 JMG treated with steroids forest plot


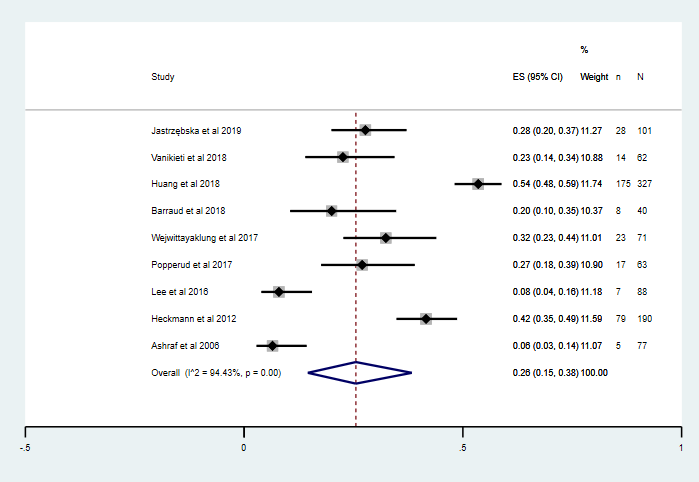


Supplementary fig. 14 JMG treated with immunosuppressants forest plot


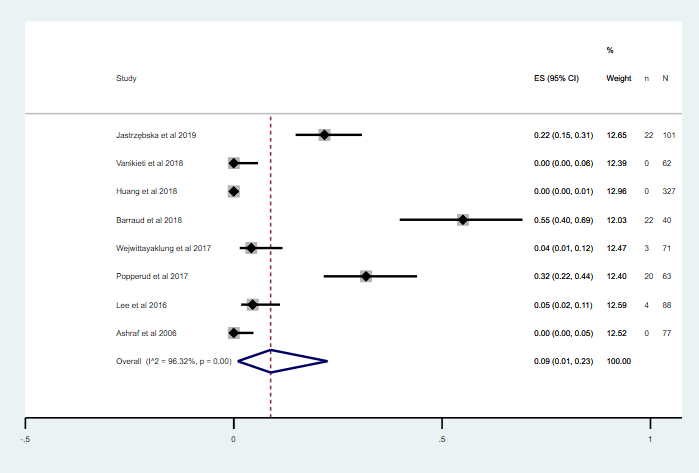


Supplementary fig. 15 JMG treated with IVIG forest plot


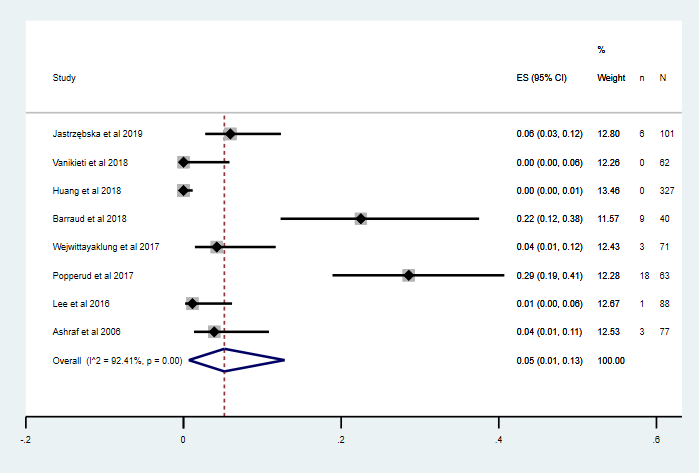


Supplementary fig. 16 JMG treated with PE forest plot


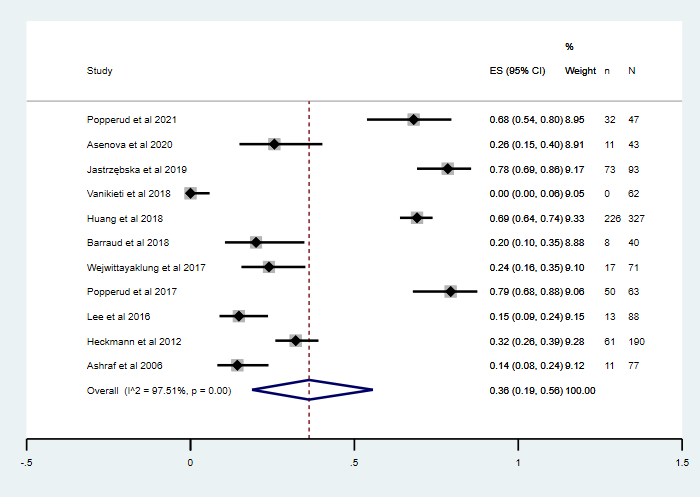


Supplementary fig. 17 JMG performed thymectomy forest plot


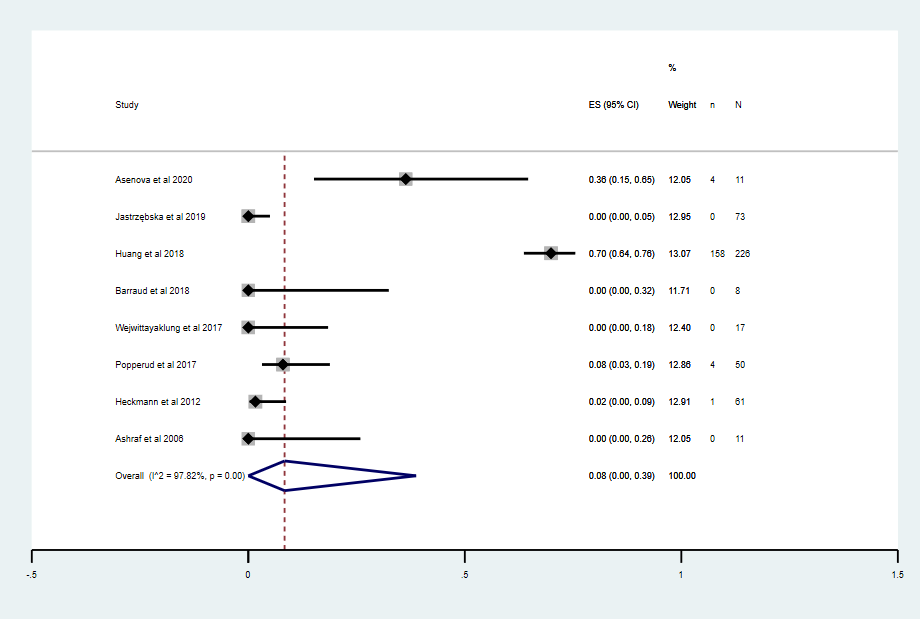


Supplementary fig. 18 JOMG performed thymectomy forest plot


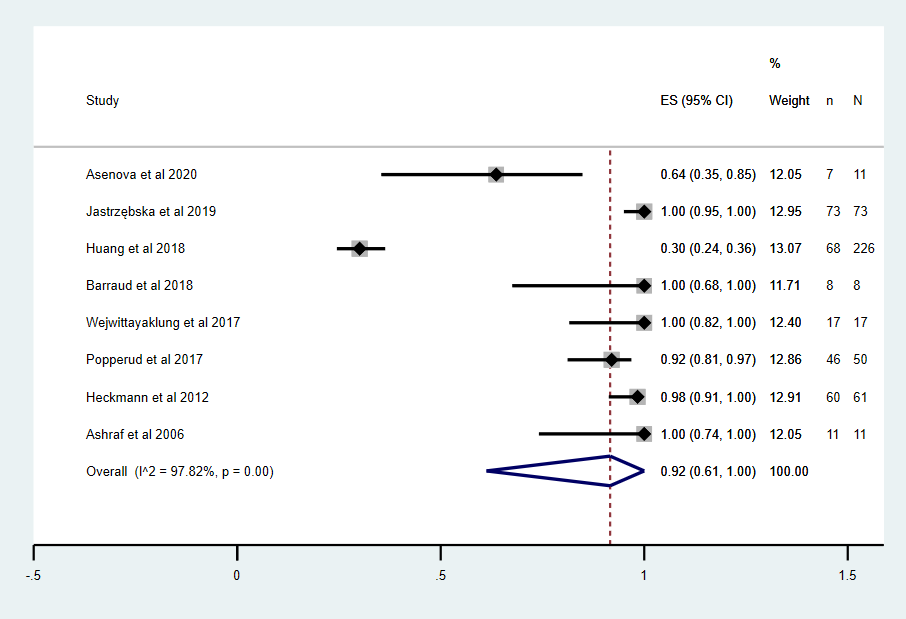


Supplementary fig. 19 JGMG performed thymectomy forest plot


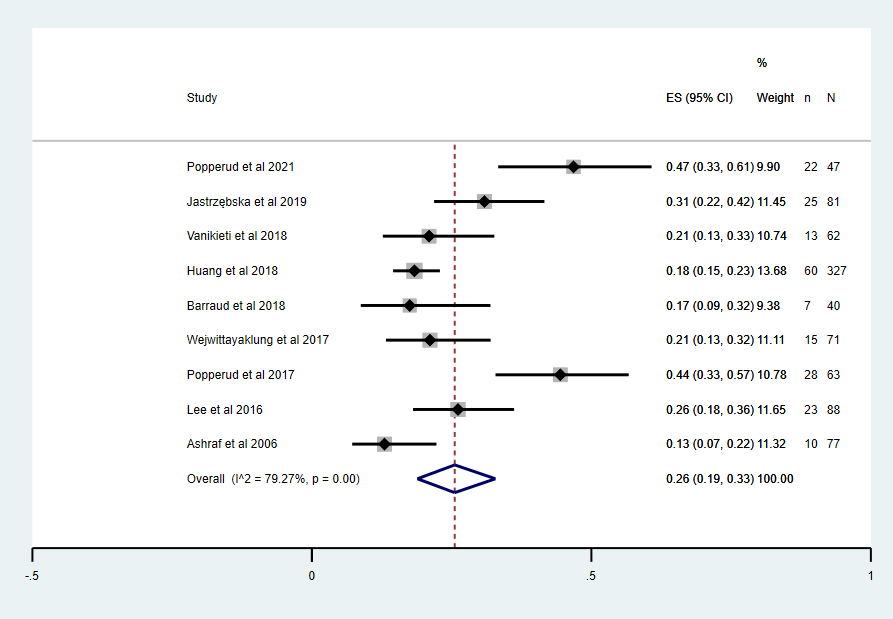


Supplementary fig. 20 JMG achieved CSR forest plot

**
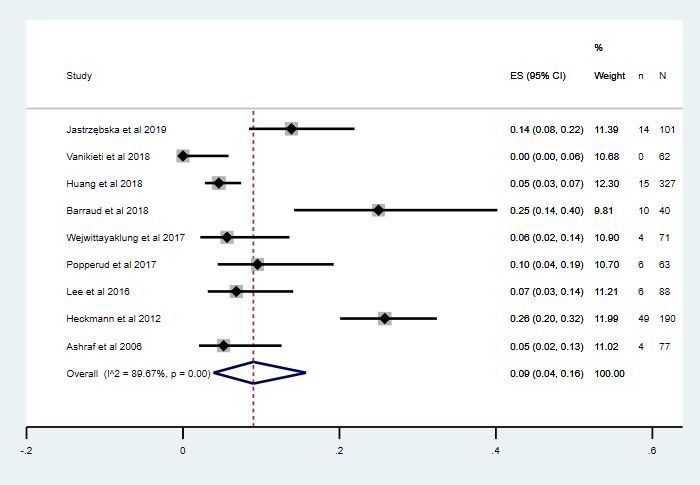
**

Supplementary fig. 21 JMG with a history of myasthenic crisis forest plot


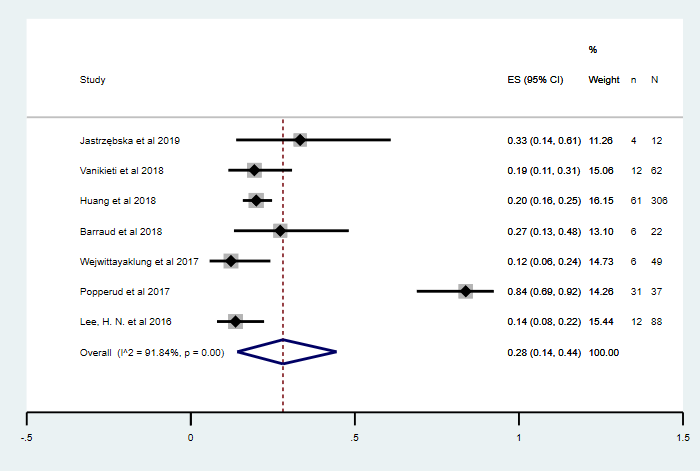


Supplementary fig. 22 JMG with generalization forest plot
